# Increased Thrombogenicity is Associated With Coronary Microvascular Dysfunction in Patients With STEMI—A Proof‐of‐Concept Study

**DOI:** 10.1002/ccd.31612

**Published:** 2025-05-27

**Authors:** Roberto Scarsini, Denis Leonardi, Andrea Bottardi, Concetta Mammone, Leonardo Portolan, Caterina Butturini, Verdiana Galli, Francesco Della Mora, Alessandro Ruzzarin, Sara Pazzi, Anna Piccoli, Simone Fezzi, Domenico Tavella, Gabriele Pesarini, Leonardo Gottin, Flavio Ribichini

**Affiliations:** ^1^ Department of Medicine, Division of Cardiology University of Verona Verona Italy; ^2^ Department of Clinical and Molecular Medicine Sapienza University Rome Italy; ^3^ Department of Surgery, Division of Anesthesia and Intensive Care Unit for Cardiac and Thoracic Surgery University of Verona Verona Italy

**Keywords:** angiography‐derived index of microcirculatory resistance, coronary microvascular dysfunction, microvascular obstruction, ST‐elevation myocardial infarction, thromboelastography

## Abstract

**Background:**

Up to 50% of patients with ST‐segment elevation myocardial infarction (STEMI) undergoing primary percutaneous coronary intervention (pPCI) develop coronary microvascular dysfunction (CMD).

**Aims:**

This study aims to assess whether a prothrombotic state of coronary blood, defined by thromboelastography (TEG), is associated with post‐pPCI CMD in STEMI.

**Methods:**

TEG analysis of infarct‐related coronary artery (IRA) blood was performed in 25 consecutive STEMI patients before and after successful pPCI. CMD was defined by high values of the angiography‐derived index of microcirculatory resistance (IMR_angio_ ≥ 40 units).

**Results:**

The median age was 59 (IQR 51−71) years. CMD was observed in 13 (52%) patients. Pre‐pPCI Reaction (R) and Kinetic (K) time of the coronary blood from the IRA were inversely correlated with IMR_angio_ (rho −0.45, *p* = 0.02; rho −0.62, *p* = 0.002 respectively). R time was significantly shorter in patients who developed CMD (10.7 [IQR 8.8, 13.1] vs. 18.2 [IQR 12.6, 24.2], *p* = 0.05), and it provided an overall good diagnostic accuracy in predicting CMD (AUC 0.75, [95% CI 0.53−0.96] *p* = 0.05). Similar relationship was founded for K time: 3.5 [IQR 2.2−4.6] versus 10.6 [IQR 4.5−30.0] min, *p* = 0.01; AUC 0.82, [95% CI 0.64−0.99] *p* = 0.01. Moreover, *α* angle was significantly larger (57.4 [IQR 46.5−64.8] vs. 40.0 [IQR 39.0−49.5], *p* = 0.03) in patients with CMD (AUC 0.78, [95% CI 0.57−0.98], *p* = 0.03). Coronary post‐pPCI TEG parameters were not associated with CMD.

**Conclusions:**

In this proof‐of‐concept study, a prothrombotic state of coronary blood from the IRA is associated with CMD. Further studies are warranted to evaluate if TEG may enhance individualized therapies in patients with STEMI at risk of CMD.

## 1 Background

Primary percutaneous coronary intervention (pPCI) after ST‐segment elevation myocardial infarction (STEMI) has reduced intrahospital mortality. However, despite successful restoration of epicardial coronary patency, no‐reflow may occur due to coronary microvascular dysfunction (CMD) [1].

CMD, identified with coronary physiology as high microcirculatory resistances is associated with a high risk of mortality and heart failure (HF) after STEMI, regardless of the modality of assessment [2, 3].

Individual intrinsic haemostatic properties may affect the risk of CMD. Thromboelastography (TEG) is a point‐of‐care whole‐blood ex vivo assay that measures the dynamic viscoelastic properties of clot generation from the time of initial plasmatic thrombin generation to platelet‐fibrin dependent clot formation, clot strengthening, and clot lysis. TEG can also provide comprehensive information on the interaction between fibrinogen/fibrin complex and platelets to form a platelet‐fibrin clot [4] and fibrinogen (level and activity) to clot formation and stability.

Previous studies have used TEG to define the degree of hypercoagulability and long‐term effects of anti‐platelets in acute and chronic coronary syndromes (ACS and CCS) [5, 6]. TEG has also been used in coronary artery disease to correlate thrombin‐induced platelet fibrin clot strength with platelet volume indices and inflammatory markers [7]. However, few studies have evaluated the impact of haemostatic components on CMD occurrence in patients with acute myocardial infarction (AMI) [8, 9]. TEG‐defined hypercoagulability, observed in pre‐pPCI peripheral arterial samples, was associated with high IMR in patients with AMI. However, whether a TEG assessment from coronary samples before and after successful pPCI predicts the risk of CMD remains unclear. In this study, we aimed to assess if increased thrombogenicity, detected in coronary blood before and after pPCI, correlated with CMD in a cohort of consecutive patients with STEMI.

## 2 Methods

### 2.1 Patient Population

Between July 2022 and December 2022, 91 consecutive patients with STEMI referred to Verona University Hospital for pPCI were considered for enrollment.

Patients with STEMI were identified and treated according to the current ESC guidelines [9].

Exclusion criteria were: age < 18 years or > 80 years, females of childbearing age, atrioventricular block, hemodynamic instability, symptoms onset prior than 12 h before, previous AMI, previous coronary artery bypass graft surgery (CABG), left main or multivessel disease (MVD) requiring cardiac surgery, ongoing anticoagulant or anti‐platelet therapy, known inherited or acquired blood disorders (such as mutation of Leiden factor, antiphospholipid syndrome, hemophilia, sepsis, disseminated intravascular coagulation [DIC]).

The local ethical committee approved the study protocol, and the study was conducted in accordance with the Declaration of Helsinki. All participants provided written informed consent.

### 2.2 Data Collection and Clinical Follow‐Up

Baseline demographic, angiographic and procedural data were collected prospectively in a dedicated electronic case report form. Patients were clinically evaluated 6, 12, 18, and 24 months after the index procedure. During index hospitalization and at 12‐month follow‐up, patients underwent transthoracic echocardiography.

The occurrence of a major adverse cardiac event (MACE), namely the composite of cardiovascular (CV) mortality, rehospitalization for HF and target vessel failure (TVF), was considered at the longest available follow‐up.

### 2.3 Medical Therapy

STEMI patients were treated according to a standardized institutional protocol. All the patients received a loading dose (LD) of aspirin (250 mg intravenous [iv]) and P2Y12 inhibitor (ticagrelor 180 mg). Unfractionated heparin (UFH) was administered in two phases: 5000 UFH units were administered through the radial access before coronary angiography. The remaining weight‐adjusted dose of UFH was administered in the IRA after coronary angiography and before pPCI. In cases of complex PCI, additional doses of UFH were administered based on Activated Clotting Time (ACT) measurements, which were routinely performed every 15 min. After pPCI, secondary prevention measures were optimized according to the latest guideline‐recommended pharmacologic therapy.

### 2.4 Laboratory Data

Baseline biochemical data, including high‐sensitivity troponin T (hs‐TnT), complete blood count, creatinine, electrolytes and coagulation profile with Prothrombin Time (PT) and activated Partial Thromboplastin Time (aPTT), were immediately collected after arrival to the emergency department according to our institutional protocol.

### 2.5 Thrombogenicity and Thrombotic Burden

Coronary angiography and pPCI were performed according to standard clinical practice from radial or femoral arterial access. After oral P2Y12 Inhibitor administration (Ticagrelor) and selective cannulation of the IRA with the 6 F guiding catheter, a 5 mL arterial blood sample from the IRA was obtained for TEG assessment, before the intracoronary remaining UFH administration.

Global haemostasis analysis was performed with Haemonetics TEG 6 s. Through sample vibration and thanks to light‐emitting‐diode illumination and a disposable multi‐channel microfluidic cartridge, TEG 6S allows a rapid assessment of haemostasis.

**Figure 1:**
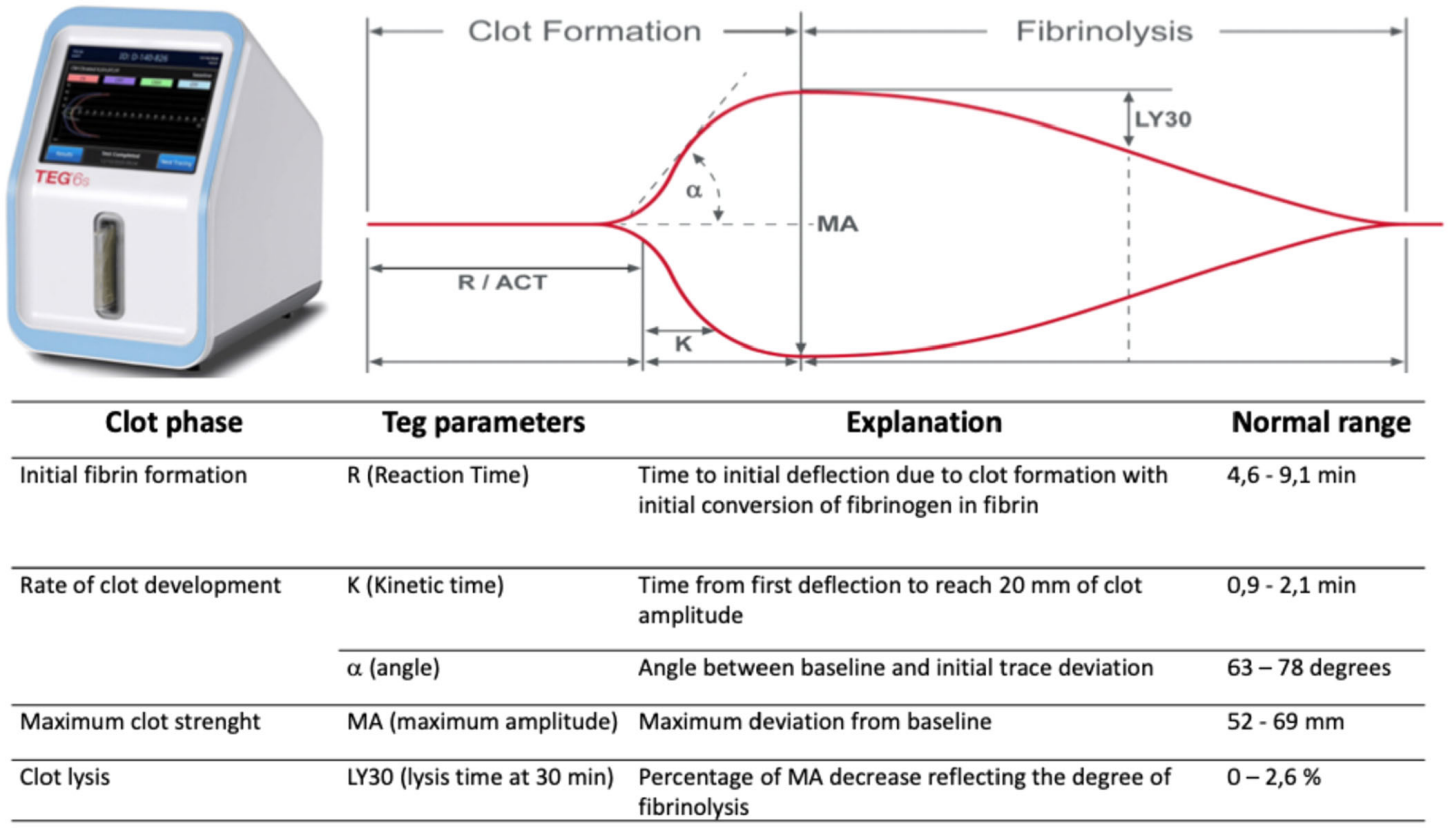
TEG parameters. [Color figure can be viewed at wileyonlinelibrary.com]

The following TEG parameters were obtained from Citrated Kaolin with Heparinase (CKH) assay (Figure 1): reaction (R) time, defined as the time to initial deflection due to clot formation with the initial conversion of fibrinogen in fibrin; kinetic (K) time, time from first deflection to reach 20 mm of clot amplitude; alpha (*α*) angle, angle between baseline and initial trace deviation; maximum amplitude (MA), defined by maximum deviation from baseline. The maximum time for R and K time analysis was set at 30 min a priori.

The TEG assessment of blood collected from the IRA was repeated at pPCI completion.

The thrombogenicity was also evaluated by assessing TIMI Thrombus grade before pPCI [10].

After pPCI, on the patient's arrival in the Coronary Care Unit, a comprehensive biochemical assessment, including glycolipid profile, N‐terminal prohormone of brain natriuretic peptide (NT‐proBNP), fibrinogen and hs‐TnT, was performed.

### 2.6 Angiography‐Derived Index of Microcirculatory Resistance (IMR_angio_)

Post‐pPCI coronary physiology indices, including quantitative flow ratio (QFR) and IMR_angio_, were measured as previously described [11]. Briefly, QFR in resting condition (QFR_rest_) was measured offline post pPCI using QAngio XA 3D software (Medis) by two independent certified operators. IMR_angio_ was assessed offline with 3‐dimensional‐vessel reconstruction and characterization of coronary blood flow based on the following formula:

$${\mathrm{IMR}}_{\mathrm{angio}}={\mathrm{Pa}}_{\mathrm{rest}}\times {\mathrm{QFR}}_{\mathrm{rest}}\times {\mathrm{nframes}}_{\mathrm{rest}}/\mathrm{FRA}$$

Pa_rest_ being the mean aortic pressure measured at rest, *n* frames_rest_ being the number of frames for contrast dye to reach distal landmarks in resting conditions and FRA being the frame acquisition rate set at 15 frames/s.

### 2.7 Cardiac Magnetic Resonance Imaging

CMR was performed in 19 (76%) patients using a 3.0‐T magnetic resonance scanner (MAGNETOM TIM Trio or MAGNETOM Verio, Siemens Healthcare, Erlangen, Germany) within 3 ± 2 days after pPCI. Cvi42 image analysis software (Circle Cardiovascular Imaging Inc., Calgary, Canada) was used for image analysis. Left and right ventricular (LV and RV) volumes, mass and ejection fraction (LVEF and RVEF) were assessed from steady‐state free precession images. Parameters included volume and mass of T2‐sequence bright signs of oedema, MVO and gray zones. To quantify the percentage of LV mass infarct size (LVMIS%), as depicted by late gadolinium enhancement (LGE), the signal intensity threshold was set at 5 SDs above the mean signal intensity of the remote reference myocardium. MVO, characterized by a no‐flow phenomenon [12], was defined as the hypointense area within the LGE region, and its size was quantified by manual delineation. To standardize MVO to LV global mass, the ratio between absolute MVO and global LV mass (MVO ratio, g/g) was provided.

### 2.8 CMD

CMD was defined as IMR_angio_ ≥ 40 U [13] in the IRA following revascularization.

### 2.9 Statistical Analysis

Categorical variables were presented as numbers and percentages and compared with the Fisher exact test. Continuous variables were presented as median values and interquartile range and compared with the Mann−Whitney *U* test. Linear regression models were performed to assess the association between variables, and the Spearman rho index was provided. The areas under the receiver‐operating characteristic (ROC) curve (AUC) were calculated to test the ability of TEG parameters to predict CMD. The rate of composite clinical endpoint in patients with and without CMD was compared with the log‐rank test.

All probability values were two‐sided, and *p* < 0.05 were considered statistically significant. Statistical analysis was performed through IBM SPSS Statistics 26.0.

## 3 Results

### 3.1 Study Cohort Characteristics

**Figure 2:**
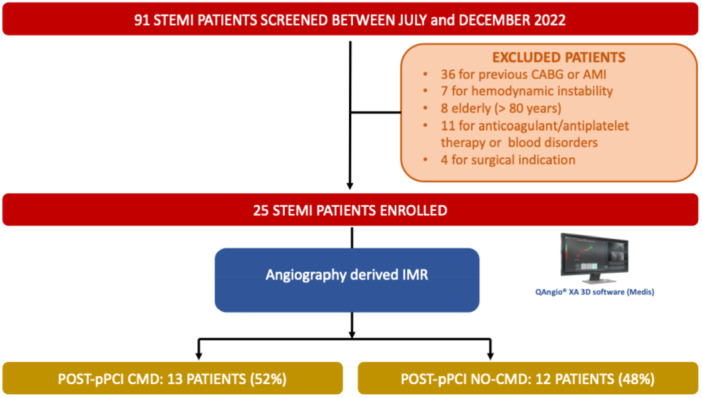
Flow‐chart to achieve the cohort of study and classification in STEMI patients without or with coronary microvascular dysfunction (CMD). AMI, acute myocardial infarction; CABG, coronary artery bypass graft surgery; pPCI, primary percutaneous coronary intervention. [Color figure can be viewed at wileyonlinelibrary.com]

Overall, 25 consecutive patients with STEMI were included in the study (Figure 2).

The mean age was 59 years (IQR 51−71), and 20 patients (80%) were male. Eleven patients (44%) were hypertensive, 3 of them (12%) had diabetes. Baseline clinical characteristics of the study cohort are reported in Table 1.

**Table 1:** Clinical and procedural characteristics.

Baseline data	Overall (*n* = 25)	No CMD (*n* = 12)	CMD (*n* = 13)	*p* value
Age (years)	59 (51, 71)	59.5 (51, 67)	57 (50, 72)	0.94
Sex (Male)	20 (80%)	6 (50%)	13 (52%)	0.58
BMI (Kg/m^2^)	25 (23, 27)	26 (24, 27)	25 (23, 30)	0.98
SBP pre pPCI (mmHg)	155 (130, 174)	160 (130, 186)	153 (130, 167)	0.37
DBP pre pPCI (mmHg)	85 (80, 96)	90 (70, 100)	85 (80, 90)	0.49
MAP pre pPCI (mmHg)	106 (97, 125)	110 (97, 127)	103 (97, 114)	0.40
SBP post pPCI (mmHg)	127 (112, 150)	138 (111, 154)	125 (115, 142)	0.79
DBP post pPCI (mmHg)	80 (70, 90)	76 (66, 89)	85 (74, 95)	0.15
MAP post pPCI (mmHg)	102 (87, 106)	97 (83, 109)	102 (90, 106)	0.46
Hypertension	11 (44%)	4 (33%)	7 (54%)	0.43
Family CAD	9 (36%)	4 (33%)	5 (38%)	1.00
Dyslipidemia	6 (24%)	5 (42%)	1 (8%)	0.07
Diabetes	3 (12%)	3 (25%)	0 (0%)	0.09
History of Smoking	18 (78%)	8 (67%)	10 (77%)	0.16
Previous MI	0 (0%)	0 (0%)	0 (0%)	—
PAD	1 (4%)	0 (0%)	1 (8%)	1
Previous stroke	1 (4%)	0 (0%)	1 (8%)	1
CKD	4 (16%)	2 (17%)	2 (15%)	1
Time in ICU (nights)	2 (2, 3)	2 (1, 2)	2 (2, 3)	0.26
Time in hospital (days)	7 (6, 8)	7 (6, 9)	7 (7, 8)	0.79
*Procedural data*				
ECG to balloon time (minutes)	78 (64, 94)	81 (65, 94)	75 (55, 105)	0.82
Patient delay (from symptoms to ECG—min)	60 (37, 100)	55 (37, 95)	68 (31, 279)	0.56
Total ischemic time (from symptoms to balloon—min)	150 (124, 220)	150 (126, 220)	155 (109, 342)	0.88
Access (radial)	25 (100%)	12 (100%)	13 (100%)	1
Culprit vessel (LAD)	16 (64%)	6 (50%)	10 (75%)	0.23
Proximal main vessel lesion	6 (24%)	3 (25%)	3 (23%)	1
TIMI flow pre pPCI	0 (0,1)	0 (0,2)	0 (0,1)	0.53
TIMI 3 post‐pPCI	24 (96%)	12 (100%)	12 (92%)	1
High visual thrombotic burden	16 (64%)	5 (42%)	11 (85%)	0.04
Thromboaspiration	13 (52%)	7 (58%)	6 (46%)	0.70
Predilation	19 (76%)	8 (67%)	11 (85%)	0.38
Number of DES per culprit vessel	1 (1,1)	1 (1,1)	1 (0,1)	0.33
Post‐dilation	15 (63%)	6 (50%)	9 (55%)	0.44
Angiographic no‐reflow after pPCI	3 (12%)	0 (0%)	3 (23%)	0.22
Multivessel disease	18 (72%)	10 (73%)	8 (62%)	0.38
PCI to nonculprit lesion	4 (16%)	2 (17%)	2 (15%)	1

Abbreviations: BMI = body mass index, CAD = coronary artery disease, CKD = chronic kidney disease, CKH = citrate‐kaolin‐heparinase, CMD = coronary microvascular dysfunction, DBP = diastolic blood pressure, MA = maximum amplitude, MAP = mean arterial pressure, PAD = peripheral artery disease, pPCI = primary percutaneous coronary intervention, SBP= systolic blood pressure.

### 3.2 Post‐Ischemic CMD

The median value of IMR_angio_ was 46 (IQR 28, 55) units. CMD defined by IMR_angio_ ≥ 40 units was present in 13 (52%) patients (Figure 2). Clinical, laboratory and echocardiographic data of patients stratified according to IMR_angio_ are reported in Tables 1, 2, 3. Post‐pPCI IMR_angio_ was significantly correlated with MVO (rho 0.62, *p* = 0.01; AUC 0.85, [95% CI 0.65−1.00] *p* = 0.02, Figure S1) and with NT‐proBNP values (rho 0.49, *p* = 0.02).

**Table 2:** Laboratory and TEG data.

Lab data	Overall (*n* = 25)	No CMD (*n* = 12)	CMD (*n* = 13)	*p* value
Haemoglobin (g/dL)	14.4 (13.3, 15.5)	14.5 (13.1, 15.8)	14.4 (13.3, 15.0)	0.53
Red blood cell number (×10^9^/L)	4.9 (4.5, 5.2)	5.0 (4.6, 5.4)	4.6 (4.2, 4.9)	0.07
Haematocrit (%)	42.0 (39.9, 45.4)	42.5 (39.6, 47.0)	41.5 (38.3, 44.4)	0.57
White blood cell number (×10^9^/L)	11.4 (10.1, 12.8)	11.8 (11.1, 12.8)	10.8 (9.3, 13.3)	0.40
White blood cell number Peak (×10^9^/L)	12.8 (10.9, 14.1)	12.4 (11.2, 13.9)	12.9 (10.7, 14.1)	0.98
Platelets (×10^9^/L)	238.0 (222.5, 269.0)	247.0 (221.2, 263.0)	237.0 (223.0, 280.0)	0.94
PT INR pre pPCI	1.0 (1.0, 1.1)	1.0 (0.9, 1.1)	1.1 (1.0, 1.1)	0.08
aPTT pre pPCI	1.0 (0.9, 1.1)	0.9 (0.8, 1.0)	1.0 (0.9, 1.2)	0.14
PT INR in ICU	1.1 (1.1, 1.2)	1.1 (1.1, 1.2)	1.2 (1.1, 1.2)	0.49
aPTT in ICU	0.9 (0.8, 1.0)	0.9 (0.9, 1.4)	0.9 (0.8, 1.0)	0.66
Fibrinogen (g/L)	3.5 (3.0, 4.5)	3.8 (3.0, 4.5)	3.5 (2.8, 5.6)	0.73
CRP Peak (mg/L)	32.0 (0.4, 10.0)	1.1 (0.5, 4.4)	9.1 (0.2, 12.5)	0.37
LDL cholesterol (mg/dL)	111.5 (94.5, 148.7)	130.0 (66.0, 158.0)	110.0 (96.0, 134.5)	0.43
HDL cholesterol (mg/dL)	41.0 (36.5, 51.5)	41.0 (34.7, 49.5)	43.0 (37.0, 52.5)	0.53
Triglycerides (mg/dL)	115.0 (77.0, 147.5)	127.0 (78.7, 156.2)	92.0 (73.0, 142.0)	0.43
Total Cholesterol (mg/dL)	179.0 (156.5, 213.0)	178.0 (144.7, 229.2)	179.0 (156.5, 208.5)	0.59
NT‐proBNP (ng/L	928.0 (520.2, 2404.0)	681.0 (514.0, 1530.0)	1692.5 (590.2, 3067.2)	0.13
hs‐TnT Peak (ng/L	4517.0 (1479.0, 8037.5)	3224.0 (1192.5, 8420.2)	4537.0 (1747.5, 11753.5)	0.42
Creatinine (mg/dL)	0.9 (0.7, 1.1)	0.9 (0.6, 1.0)	0.8 (0.7, 1.2)	0.91
eGFR (ml/min/1.73m2)	98.1 (75.2, 115.7)	99.1 (77.6, 113.7)	91.1 (69.8, 117.4)	0.79

Thromboelastography data				
Coronary pre‐pPCI TEG	Overall (*n* = 25)	No CMD (*n* = 12)	CMD (*n* = 13)	*p* value
*R* time (min)	13.1 (8.9, 21.8)	18.2 (12.6, 24.2)	10.7 (8.8, 13.1)	0.05
*K* time (min)	4.5 (3.0, 12.5)	10.6 (4.5, 30.0)	3.5 (2.2, 4.6)	0.01
*α* angle (grades)	48.4 (39.0, 58.9)	40.0 (39.0, 49.5)	57.4 (46.5, 64.8)	0.03
MA (mm)	28.1 (0.0, 58.9)	10.4 (0.0, 47.9)	52.5 (12.7, 61.4)	0.09
Coronary post‐pPCI TEG				
*R* time (min)	18.4 (11.4, 21.5)	15.4 (11.2, 27.5)	19.4 (14.4, 21.2)	0.47
*K* time (min)	5.5 (2.7, 11.6)	3.3 (2.3, 11.1)	6.3 (3.1, 12.3)	0.48
*α* angle (grades)	40.5 (38.2, 55.8)	54.4 (39.0, 58.2)	40.0 (38.0, 48.1)	0.23
MA (mm)	37.9 (14.6, 59.6)	47.6 (4.8, 59.8)	33.3 (14.6, 56.3)	0.64

Abbreviations: aPTT = activated partial thromboplastin time, CMD = coronary microvascular dysfunction, CRP = C‐reactive protein, eGFR = estimated glomerular filtration rate, HDL = high‐density lipoprotein, hs‐TnT = high‐sensitivity Troponin‐T, LDL = low‐density lipoprotein, PT INR = prothrombin time international normalized ratio.

**Table 3:** Echocardiographic data.

Echo parameters	Overall (*n* = 25)	No CMD (*n* = 12)	CMD (*n* = 13)	*p* value
LV EF (%)	47 (42, 54)	50 (45, 55)	42 (39, 52)	0.08
LVEDVi (mL/m^2^)	60 (53, 70)	58 (50, 61)	64 (57, 72)	0.06
LAVi (mL/m^2^)	28 (23, 33)	29 (23, 33)	27 (22, 34)	0.74
sPAP (mmHg)	30 (23, 36)	30 (23, 39)	29 (21, 34)	0.47
TAPSE	21 (19, 22)	21 (18, 24)	21 (20, 22)	0.86

Abbreviations: CMD = coronary microvascular dysfunction, LAVi = left atrial volume indexed, LVEDVi = left ventricle end‐diastolic volume indexed, LV EF = left ventricle ejection fraction, sPAP = systolic pulmonary arterial pressure, TAPSE = tricuspid annular plane excursion.

### 3.3 Thrombogenicity and Myocardial Injury

**Figure 3:**
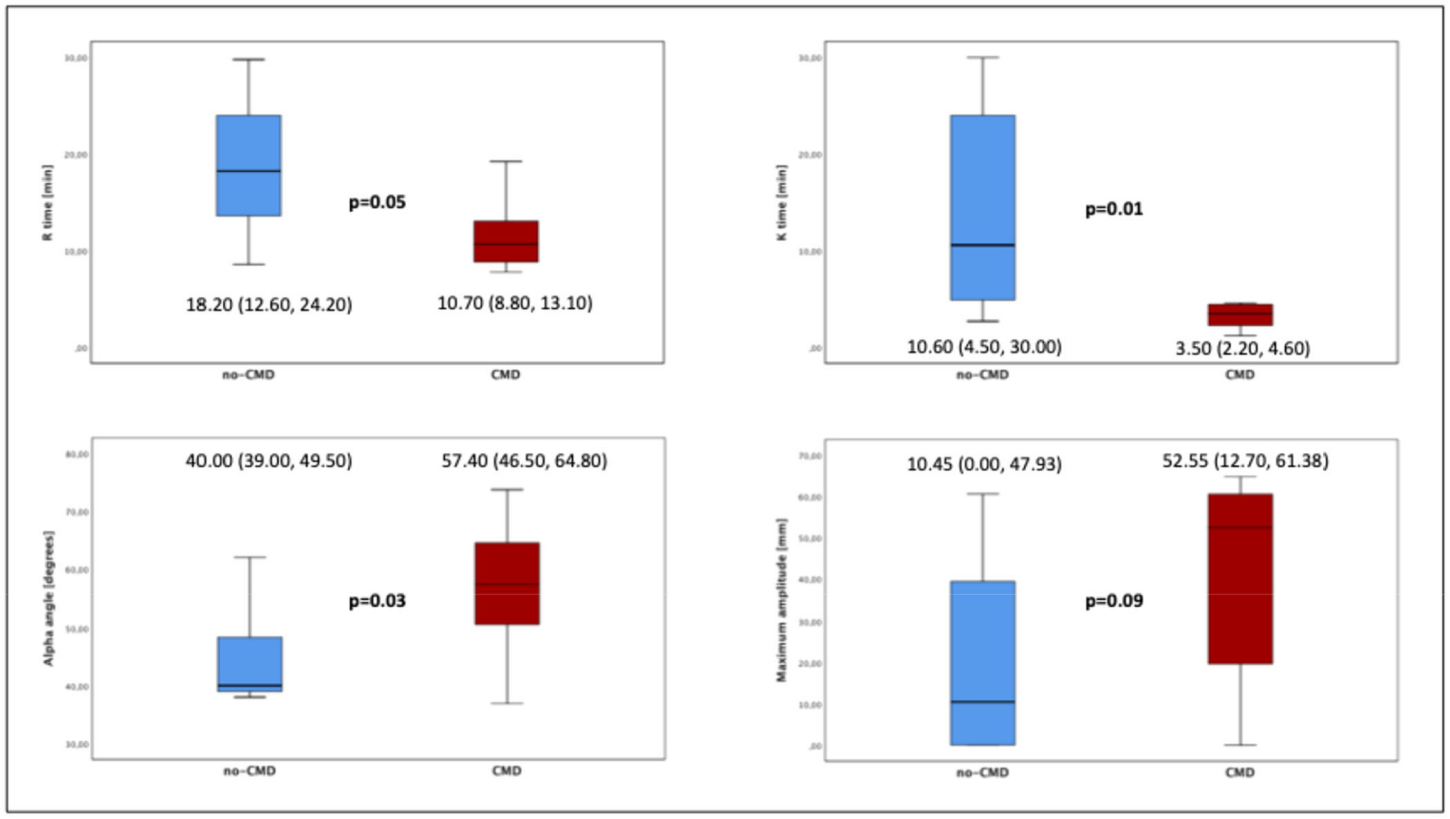
Pre‐PCI TEG parameters patients with and without CMD. *R* time, *K* time, alpha angle, maximum amplitude. [Color figure can be viewed at wileyonlinelibrary.com]

Patients with CMD showed shorter *R* time 10.7 [IQR 8.8, 13.1] versus 18.2 [IQR 12.6, 24.2], *p* = 0.05) and *K* time (3.5 [IQR 2.2–4.6] vs. 10.6 [IQR 4.5–30.0] minutes, *p* = 0.01) (Figure 3). *α* angle was significantly larger (57.4 [IQR 46.5–64.8] vs. 40.0 [IQR 39.0–49.5], *p* = 0.03) and MA was numerically higher larger in patients with CMD (52.5 [IQR 12.7, 61.4] vs. 10.4 [0.0 47.9], *p* = 0.09).

Pre‐pPCI *R* time (AUC 0.75, [95% CI 0.53−0.96] *p* = 0.05), *K* time (AUC 0.82, [95% CI 0.64−0.99] *p* = 0.01) and *α* angle (AUC 0.78, [95% CI 0.57−0.98], *p* = 0.03) demonstrated good accuracy in predicting CMD (Figure S2).

Conversely, post‐pPCI TEG parameters were not statistically associated with CMD (Table 2).

Pre‐PCI TEG parameters showed a significant correlation with the extent of myocardial injury, as reflected by peak hs‐TnT levels: higher TnT peak was associated with shorter *R* time (rho = 0.63, *p* = 0.003) and K time (rho = 0.61, *p* = 0.003), as well as with larger *α* angle (rho = 0.60, *p* = 0.003) and greater MA (rho = 0.66, *p* = 0.006).

### 3.4 Thrombus Burden and CMD

When patients were stratified by TIMI Thrombus grade before pPCI (grades 4–5 as high burden vs. lower grades), post‐pPCI IMR_angio_ was significantly higher in the high thrombotic burden group (*p* = 0.002, Figure S3). Notably, in this group post‐pPCI IMR_angio_ ≥ 40 U was more frequently observed (85% vs. 42%, *p* = 0.04), further supporting the link between thrombogenicity and CMD.

### 3.5 Clinical Outcomes

At a median time of 373 [IQR 329, 468] days of follow‐up, a MACE occurred in 3 (12%) patients. Among the adverse events the following were reported: re‐hospitalization for heart‐failure (HF) (*n* = 1) and TVF (*n* = 2). Adverse events occurred only in patients with CMD.

## 4 Discussion

This proof‐of‐concept study aimed to assess the TEG‐defined thrombogenicity of coronary blood and its possible correlation with the development of CMD in consecutive patients with STEMI undergoing pPCI. The main findings of our study are as follows:

1. Pre‐pPCI TEG parameters obtained from the IRA were significantly associated with hs‐TnT peak values, suggesting their potential role as markers of the extent of myocardial injury.
2. Pre‐pPCI TEG parameters obtained from the IRA emerged as significant predictors of CMD.
3. Conversely, post‐pPCI TEG assessment was not associated with CMD, potentially indicating that the development of coronary microvascular impairment is an early phenomenon in the pathophysiology of the IRA, and its impact on myocardial reperfusion may be independent of epicardial patency restoration.

Post‐ischemic CMD recognizes complex pathophysiological mechanisms involving ischemia‐related injury, reperfusion‐related injury, and distal embolization acting on a pre‐existing substrate of an individual's increased susceptibility. The inadequate supply of coronary blood, the imbalance of vasodilator and vasoconstrictor mediators, and the ischemia‐induced swelling of the endothelial cells have a notable role in inducing CMD. The subsequent flow restoration may paradoxically add further damage related to oxygen free radicals release and the formation of neutrophil–platelet aggregates. Moreover, distal embolization of the coronary thrombus and the fissured plaque components contribute to the further worsening of the CMD as they aggravate the MVO. Arterioles and small capillaries are particularly sensitive to these flow and metabolic changes [14].

The assessment of coronary microcirculatory resistances has been repeatedly associated with clinically relevant CMD [15]. An IMR_angio_ ≥ 40 has been associated with poorer clinical outcomes, MVO and impaired residual LVEF at follow‐up [3, 16, 17].

Moreover, MVO is a well‐established and validated tool for detecting CMD. MVO is a major determinant of the infarct size and left ventricle remodeling, relating to the clinical outcomes, including hospital admissions for HF [18].

Biomarkers such as NT‐proBNP and hs‐TnT are also good indicators of CMD, both in ischemic and non‐ischemic settings [19, 20].

Similarly [13, 14, 15], our observations corroborate those previous findings.

### 4.1 Prothrombotic State and CMD

In this study, shorter K time and R time and larger α angle in pre‐pPCI coronary blood samples from the IRA are associated with higher IMR_angio_ values. The utility of TEG‐defined hypercoagulability status in ACS is emerging. In a recent study, Lee SH. and colleagues showed that patients with AMI had higher platelet‐fibrin clot strength (PFCS), measured with MA, and lower fibrinolytic activity (measured with LY30) in preprocedural arterial samples as compared to non‐AMI patients (MA 66.5 ± 7.8 vs. 65.3 ± 7.2 mm, *p* < 0.001; LY30 0.9 ± 1.8% vs. 1.1 ± 1.9%, *p* < 0.001, respectively). They also observed higher levels of PFCS (MA 67.1 ± 7.4 vs. 65.8 ± 8.3 mm, *p* < 0.001) in STEMI vs. non‐STEMI patients. Overall, hypercoagulability (i.e., MA ≥ 68 mm) was found to be more common in patients with AMI compared with non‐AMI patients (respectively: 44.9% vs. 35.4%, *p* < 0.001% and 53.7% vs. 42.9%, *p* < 0.001). The combination of hypercoagulability and impaired fibrinolysis pinpointed a subgroup of patients with a tendency to thrombogenicity and an increased risk of 4‐years MACE 31.2% versus 10.7%; adjusted hazard ratio [HR] 1.78, 95% confidence interval [CI] 1.13–2.81; *p* = 0.01) when compared with those with normal coagulability and normal fibrinolytic activity (MA < 68 mm and LY30 ≥ 0.2%) [21]. In another study by Kang et al. MA ≥ 68 mm in pre‐procedural arterial blood samples was found to be associated with a fourfold increase in the risk of having IMR > 40 U after AMI, indicating a strong association between intrinsic hypercoagulability and CMD. Moreover, they reported a higher risk of MACE occurrence in patients with both high IMR and high PFCS (HR 5.58, 95% CI, 1.31 to 23.68; *p* = 0.02) [8].

The role of thrombotic profile to predict CMD was demonstrated also with reflectance spectral characterization of coronary thrombi [22]. This evidence further supports the role of TEG analysis of the IRA as a tool to spot subjects at risk of CMD, and it identifies thrombogenicity as a potential therapeutic target in patients with AMI. However, the best strategy to reduce the risk of CMD in patients with increased thrombogenicity is far from being determined. De Maria et al. demonstrated that, in the presence of a high thrombotic burden, stent implantation may exacerbate CMD through distal embolization, compared to cases with a lower thrombotic profile [23]. Percutaneous coronary revascularization with repeated balloon dilation cannot prevent distal microembolization, which is induced and maintained by platelet activation. Glycoprotein (GP) IIb/IIIA inhibitors are currently not recommended in routine treatment of ACS patients, given the absence of solid evidence for any additional benefit of IV administration [24, 25]. Nevertheless, direct intracoronary administration of GP IIb/IIIa inhibitors during pPCI has been proposed to enhance coronary flow and potentially reduce CMI. Ma et al. demonstrated that intracoronary tirofiban administration in STEMI patients undergoing pPCI provided a significant benefit in decreasing the occurrence of MVO and improving the adverse LV remodeling at 6 months compared to IV tirofiban. However, they did not find a statistically significant benefit in the occurrence of MACE during 1‐year follow‐up compared to IV administration [25]. On the other hand, despite promising results in previous studies [26], adjunctive fibrinolytic therapy in patients undergoing pPCI for STEMI did not reduce the risk of CMD. Indeed, McCartney et al. recently showed that low‐dose intracoronary administration of fibrinolytic during pPCI failed to reduce MVO in patients with STEMI [27].

Thrombus aspiration may prevent distal embolization but it did not demonstrate to improve microvascular perfusion [28]. In major randomized clinical trials (RCTs), there was no clinical benefit of Thrombus Aspiration in STEMI considering MACE rate, cardiac death and all‐cause mortality [29, 30]. However, a metanalysis including 43 RCTs showed lower rate of MACE, AMI, death and target vessel repeat revascularization comparing with thrombus aspiration compared with standard pPCI, at the cost of increased rate stroke [31].

The lack of association between post‐PCI TEG parameters and CMD may be attributed to procedural variability and the confounding effects of anticoagulation, reinforcing the hypothesis that microvascular dysfunction is primarily driven by early ischemic and reperfusion‐related events. De Maria et al. previously demonstrated significant heterogeneity in the microvascular response to stenting. Notably, stenting is a procedural step closely linked to distal embolization; in the presence of a high thrombotic burden and the need for large stent volumes, the risk of extensive microvascular injury increases [23]. This finding indirectly underscores the importance of thrombogenicity assessment before PCI, as it may guide procedural optimization in selected patients. Individuals with high thrombotic burden and elevated thrombogenicity could potentially benefit from adjunctive strategies such as intensified thrombus aspiration, use of GPIIb/IIIa inhibitors, or even a deferred stenting approach.

Further prospective studies are warranted to evaluate the efficacy of these tailored strategies in patients identified as high risk based on angiographic and TEG‐derived markers of thrombogenicity.

### 4.2 Limitations

This is a proof‐of‐concept study with several limitations. First, this is a single‐center study with a small sample size. Therefore, our findings must be interpreted as hypothesis‐generating. Second, despite CMR‐MVO was used to validate IMR_angio_ as a predictor of CMD, data were not available in all the patients.

Direct physiological assessment of microvascular function with invasive thermodilution‐based IMR was not performed. Although IMRangio has been validated in STEMI and provides reliable prognostic information, the absence of invasive confirmation remains a study limitation [32].

Post‐PCI TEG parameters may have been affected by procedural factors and variable heparin exposure, limiting their reliability in assessing thrombogenicity after revascularization.

Endothelial function was not assessed in this study due to the emergency setting of STEMI and the potential confounding effects of inflammation and thrombosis. This limits insight into the mechanisms linking CMD and thrombogenicity.

Finally, although our findings highlight a significant association between thrombogenicity and CMD, the present study design does not allow us to establish a causal relationship. It remains unclear whether increased thrombogenicity contributes to CMD development or if CMD itself leads to enhanced thrombogenicity. This bidirectional uncertainty should be addressed in future prospective studies.

## 5 Conclusions

In this proof‐of‐concept study, increased thrombogenicity in the infarct‐related artery of patients with STEMI was associated with CMD.

## Conflicts of Interest

The authors declare no conflicts of interest.

## Supporting information


**Supporting Figure 1.** On the left, median angio‐derived IMR in patients with and without MVO. On the right, angio‐derived IMR demonstrated excellent accuracy in predicting MVO with an AUC of 0.85 [95% CI 0.65 – 1.00].

**Supporting Figure 2.** TEG parameters in predicting CMD: R time, K time, alpha angle, maximum amplitude. R time: AUC 0.75, [95% CI 0.53 – 0.96] p=0.05. K time: AUC 0.82, [95% CI 0.64 – 0.99] p=0.01. Alpha angle: AUC 0.78, [95% CI 0.57 – 0.98] p=0.03. Maximum amplitude: AUC 0.75, [95% CI 0.50 – 0.99] p=0.09.

**Supporting Figure 3.** On the left, median angio‐derived IMR in patients with low and high angiographic thrombotic burden. On the right, angiographic thrombotic burden demonstrated fair accuracy in predicting CMD with an AUC of 0.72 [95% CI 0.51 – 0.93].

**Supporting** Table.

## Data Availability

The authors have nothing to report.
