# Supplementary figures and images for: Increased Thrombogenicity is Associated With Coronary Microvascular Dysfunction in Patients With STEMI—A Proof‐of‐Concept Study
Source: Catheter Cardiovasc Interv. 2025 May 27;106(2):836–45. doi: 10.1002/ccd.31612 (PMC12336792; doi:10.1002/ccd.31612)

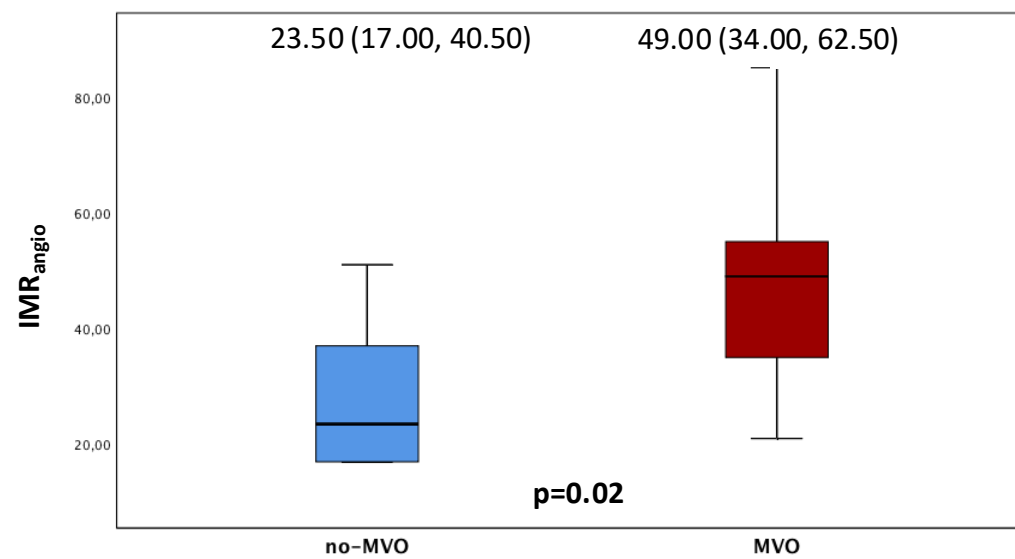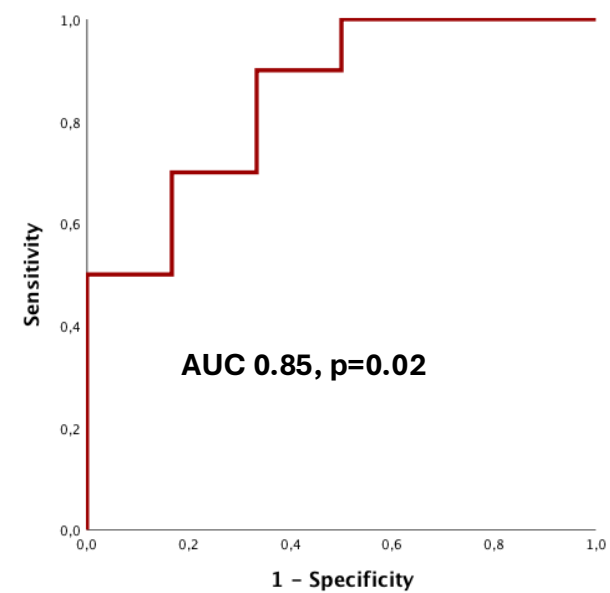

Supplement: Supplementary file 1 — Supporting Figure 1. On the left, median angio‐derived IMR in patients with and without MVO. On the right, angio‐derived IMR demonstrated excellent accuracy in predicting MVO with an AUC of 0.85 [95% CI 0.65 – 1.00]. [file CCD-106-836-s001.pdf]

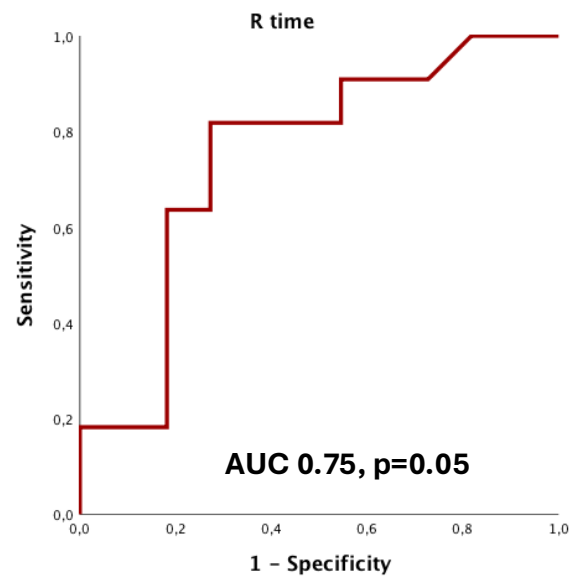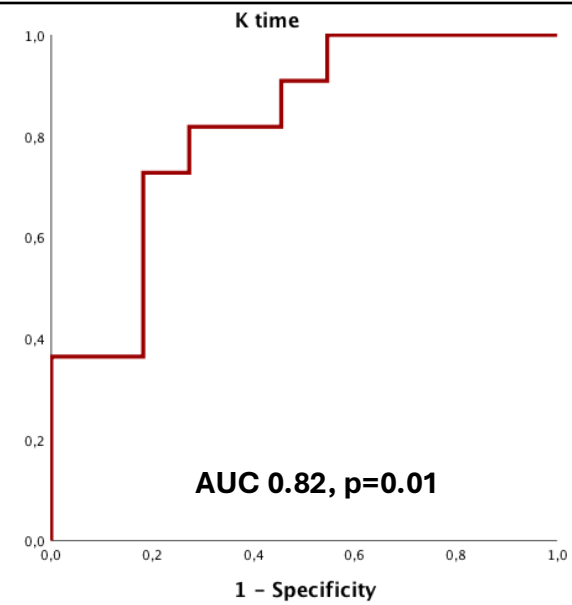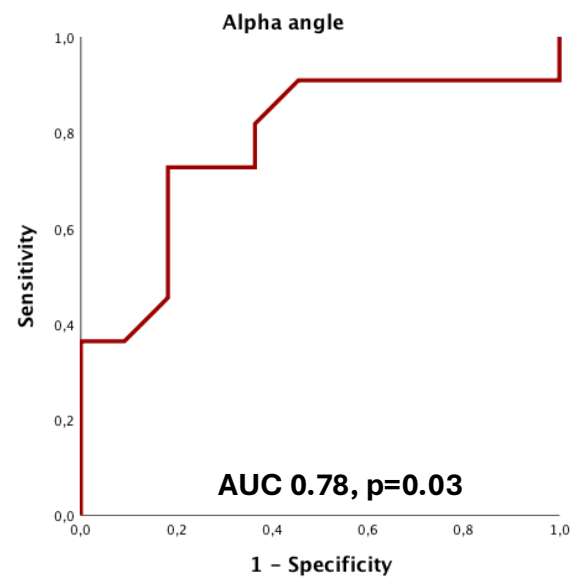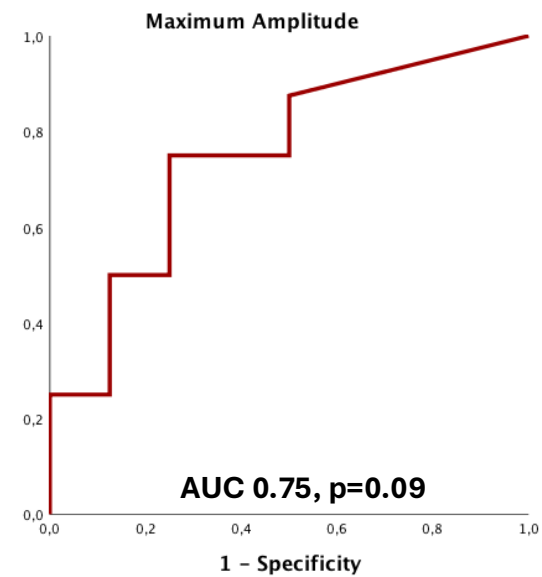

Supplement: Supplementary file 2 — Supporting Figure 2. TEG parameters in predicting CMD: R time, K time, alpha angle, maximum amplitude. R time: AUC 0.75, [95% CI 0.53 – 0.96] p=0.05. K time: AUC 0.82, [95% CI 0.64 – 0.99] p=0.01. Alpha angle: AUC 0.78, [95% CI 0.57 – 0.98] p=0.03. Maximum amplitude: AUC 0.75, [95% CI 0.50 – 0.99] p=0.09. [file CCD-106-836-s002.pdf]

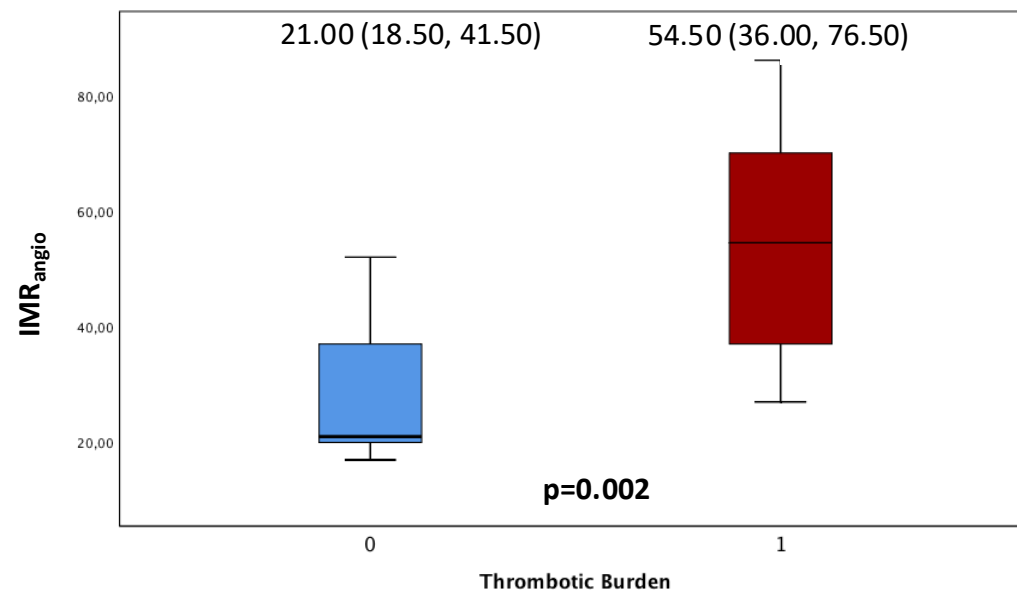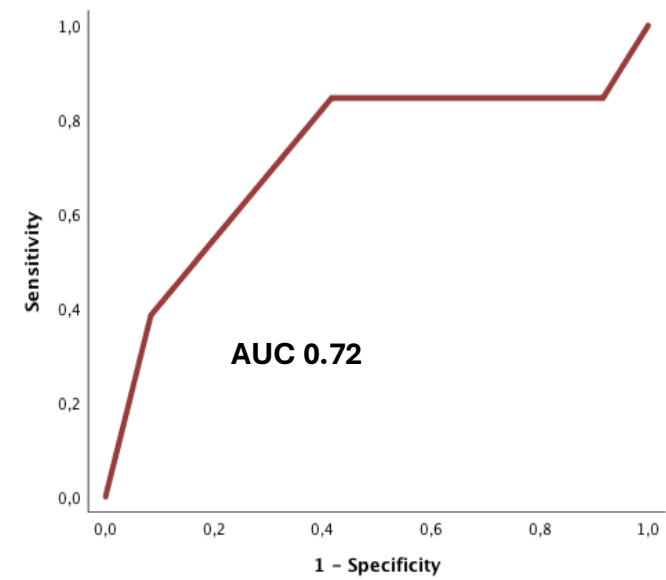

Supplement: Supplementary file 3 — Supporting Figure 3. On the left, median angio‐derived IMR in patients with low and high angiographic thrombotic burden. On the right, angiographic thrombotic burden demonstrated fair accuracy in predicting CMD with an AUC of 0.72 [95% CI 0.51 – 0.93]. [file CCD-106-836-s003.pdf]
