# Supplementary material for: Increased Thrombogenicity is Associated With Coronary Microvascular Dysfunction in Patients With STEMI—A Proof‐of‐Concept Study
Source: Catheter Cardiovasc Interv. 2025 May 27;106(2):836–45. doi: 10.1002/ccd.31612 (PMC12336792; doi:10.1002/ccd.31612)
Supplement: Supplementary file 4 — Supporting Table. [file CCD-106-836-s004.docx]

**Supplementary Table.**

| **Cardiac imaging** | | | | |
| --- | --- | --- | --- | --- |
| **CMR Parameters** | **Overall** | **No CMD** | **CMD** | **P value** |
| Left atrial end systolic area (cm^2^) | 20.6 (15.7, 23.7) | 21.1 (17.8, 25.7) | 20.0 (13.1, 24.0) | 0.22 |
| LVEDVi (ml/m^2^) | 78.4 (69.2, 91.8) | 73.8 (67.0, 92.9) | 86.3 (70.7, 92.9) | 0.49 |
| LV EF (%) | 50.8 (46.0, 54.0) | 53.6 (47.5, 57.1) | 46.6 (44.6, 52.3) | 0.05 |
| Cardiac index (ml/min/m^2^) | 2.5 (2.3, 3.0) | 2.6 (2.4, 2.9) | 2.4 (2.0, 3.2) | 0.40 |
| RVEDVi (ml/m^2^) | 66.6 (56.2, 79.8) | 70.3 (56.3, 81.2) | 59.6 (56.0, 79.4) | 0.87 |
| RV EF (%) | 56.0 (53.3, 64.4) | 58.6 (55.6, 65.3) | 53.3 (51.7, 55.5) | 0.02 |
| LGE volume (ml) | 21.0 (17.6, 31.9) | 22.8 (18.3, 33.8) | 20.5 (10.8, 23.2) | 0.49 |
| T2 volume (ml) | 146.0 (103.1, 160.8) | 126.7 (104.7, 152.0) | 160.4 (87.1, 162.5) | 0.27 |
| T2 ratio (%) | 24.6 (15.4, 32.5) | 31.5 (6.9, 38.1) | 21.1 (18.1, 29.6) | 0.56 |
| MVO (%) | 0.2 (0.0, 1.4) | 0 (0.0, 0.5) | 1.3 (0.1, 1.8) | 0.06 |

*CMD = Coronary Microvascular Dysfunction, LGE = Late Gadolinium Enhancement, LVEDVi = Left Ventricle End-Diastolic Volume indexed, LV EF= Left Ventricle Ejection Fraction, MVO = Microvascular Obstruction, RVEDVi = Right Ventricle End-Diastolic Volume indexed, RV EF = Right Ventricle Ejection fraction.*
